# Clinical factors associated with a positive postpartum depression screen in people with cardiac disease during pregnancy

**DOI:** 10.46439/Psychiatry.2.027

**Published:** 2022

**Authors:** Danielle M. Panelli, Elizabeth B. Sherwin, Christine J. Lee, Stephanie A. Leonard, Sarah E. Miller, Hayley E. Miller, Alisha T. Tolani, Valerie Hoover, Jessica R. Ansari, Abha Khandelwal, Katherine Bianco

**Affiliations:** 1 Division of Maternal-Fetal Medicine and Obstetrics, Department of Obstetrics and Gynecology, Stanford University School of Medicine, Stanford, CA, USA; 2 Department of Cardiovascular Medicine, Stanford University School of Medicine, Stanford, CA, USA; 3 Department of Psychiatry and Behavioral Sciences, Stanford University School of Medicine, Stanford, CA, USA; 4 Division of Obstetric Anesthesia, Department of Anesthesiology, Perioperative and Pain Medicine, Stanford University School of Medicine, Stanford, CA, USA

**Keywords:** Postpartum depression, Maternal cardiac disease, Pregnancy

## Abstract

**Background:**

While people with cardiac disease are known to be at increased lifetime risk of depression, little is known about postpartum depression rates in this population. Describing rates of positive postpartum depression screens and identifying risk factors that are unique to cardiac patients may help inform risk reduction strategies.

**Methods:**

This retrospective cohort study included pregnant patients with congenital and/or acquired cardiac disease who delivered at a single institution between 2014 and 2020. The primary outcome was a positive postpartum depression screen, defined as Edinburgh Postpartum Depression Score (EPDS) ≥10. Potential exposures were selected *a priori* and compared between patients with and without a positive postpartum depression screen using Wilcoxon rank-sum and Fisher’s exact tests. Secondary outcomes were responses to a longitudinal follow-up survey sent to English-speaking patients evaluating cardiac status, mental health, and infant development.

**Results:**

Of 126 eligible cardiac patients, 23 (18.3%) had a positive postpartum depression screen. Patients with a positive postpartum depression screen were more likely to have had antepartum anticoagulation with heparin or enoxaparin (56.5% versus 26.2%, p=0.007), blood transfusion during delivery (8.7% versus 0%, p=0.032), and maternal-infant separation postpartum (52.2% versus 28.2%, p=0.047) compared to patients with a negative screen. Among 29 patients with a positive screen who responded to the follow up survey, 50% reported being formally diagnosed with anxiety or depression and 33.3% reported child development problems.

**Conclusions:**

Our results highlight the importance of screening for postpartum depression in patients with cardiac disease, especially those requiring antepartum anticoagulation or maternal-infant separation postpartum.

## Introduction

Prenatal care for patients with cardiac disease is complex, often involving close surveillance, intensive monitoring, and peripartum interventions that can result in mother infant separation after birth [1,2]. Though these experiences can be distressing for patients, little is known about postpartum depression rates in this high-risk population. Characterizing postpartum depression and identifying risk factors might inform risk reduction strategies. Therefore, we sought to conduct an exploratory study to evaluate clinical factors associated with a positive postpartum depression screen in pregnant people with cardiac disease.

## Method

This was a retrospective cohort of people with congenital and/or acquired cardiac disease during pregnancy who delivered at a single quaternary institution between 2014 and 2020. People with cardiac disease who had Edinburgh Postpartum Depression Scores (EPDS) documented at the postpartum visit – which typically occurs 4–6 weeks after delivery – were included. If patients had more than one pregnancy, only the first pregnancy at our institution with an EPDS score was included. The primary outcome was a positive depression screen, defined as EPDS score ≥10 [3]. Potential exposures associated with a positive postpartum depression screen were selected *a priori* based on prior literature and included: composites of adverse maternal cardiac and neonatal events; measures of cardiac disease severity, such as World Health Organization (WHO) Classification at the beginning of pregnancy or anticoagulation with heparin or enoxaparin; substance use; delivery complications such as blood transfusion or postpartum readmission; breastfeeding status at hospital discharge; and mother-infant separation during the delivery hospitalization due to level of care (e.g. maternal or neonatal ICU admission).

Secondary outcomes were responses to a follow-up survey that was sent to all English speaking patients who provided contact information and consented to future research. This survey included questions about cardiac status, mental health, and infant development since the index delivery. Wilcoxon rank-sum and Fisher’s exact tests were used to compare clinical factors between patients with and without positive postpartum depression screens.

## Results

Of 126 eligible cardiac patients, 23 (18.3%) had a positive postpartum depression screen. Patients with a positive postpartum depression screen were more likely to have had antepartum anticoagulation with heparin or enoxaparin (56.5% versus 26.2%, p=0.007), blood transfusion (8.7% versus 0%, p=0.032), and maternal-infant postpartum separation (52.2% versus 28.2%, p=0.047, Table 1). There were trends toward higher-risk features in the positive postpartum depression screen group based on higher WHO scores, more antepartum hospitalizations, and greater preterm birth and preeclampsia rates, though these were not significantly different. Although there was a high prevalence of pre-pregnancy depression or anxiety in the whole cohort (N=26, 20.6%), there was no significant difference in positive postpartum depression screens for these patients (17.4% versus 21.4%, p=0.78).

Of the 126 patients included in this study, 71 were eligible for the follow-up survey and 29 responded (41% response rate). Among patients with a positive postpartum depression screen who responded to the survey, the median time from delivery was 1.8 years (Table 2). On the survey, 50% of patients with a positive postpartum depression screen reported being formally diagnosed with anxiety or depression and 33.3% reported child development problems such as speech delays.

## Discussion

18.3% of patients in this cohort with cardiac disease in pregnancy had a positive postpartum depression screen. For context, the prevalence of depression in the general postpartum population has been estimated between 6-13% [4,5], while the lifetime prevalence of depression in non-pregnant adults with cardiac disease is closer to 30% [6]. Interestingly, 20.6% of all people in this cohort had a pre-pregnancy diagnosis of depression or anxiety but they were not more likely to have had a positive postpartum depression screen. The majority of patients with a positive postpartum depression screen therefore did not have a prior psychiatric diagnosis; taken together, this means that 45 (35.7%) of all pregnant people with cardiac disease in this cohort had either pre-pregnancy depression or anxiety or a positive postpartum depression screen. This highlights the significance of future research to understand risk factors for depression in this population, as identification of actionable risk factors may help reduce the mental health burden these patients are facing.

A positive postpartum depression screen was significantly associated with antepartum anticoagulation with heparin or enoxaparin, blood transfusion, and maternal-infant separation during the delivery admission. The blood transfusion association should be interpreted with caution given small frequencies. Our results suggest that high risk delivery features, particularly those that result in maternal-infant separation in the immediate postpartum period, set the stage for patients with cardiac disease to develop postpartum depression. It is also notable that half of the people with a positive postpartum depression screen in this study who responded to a follow up survey reported ultimately receiving a formal diagnosis of anxiety or depression after delivery.

Strengths of this study include granular patient-level data and breadth of clinical exposures potentially associated with postpartum depression. Despite these strengths, our results must be interpreted in the context of our study design. Although this cohort represents 7 years of consecutive data from a single institution, our small sample size precludes additional analyses to adjust for potential confounders. Furthermore, findings from a single academic institution may not be generalizable to other populations. That being said, the results from our study can be used as pilot data for future research among larger, more generalizable cohorts to further examine the associations we identified.

Our results highlight the importance of screening for postpartum depression in patients with cardiac disease, especially those requiring antepartum anticoagulation or maternal-infant separation. While our study is hypothesis generating, these clinical factors warrant further investigation in larger cohorts. Addressing maternal-infant separation in particular may require operational planning, particularly for large hospital systems with separate obstetric, pediatric, and/or adult facilities.

## Figures and Tables

**Table 1:** Clinical factors compared between patients with cardiac disease who had a negative versus positive postpartum depression screen, 2014–2020.

Clinical factor	Whole cohort N= 126^a^	Negative depression screen N=103	Positive depression screen N=23	P-value^b^
**Maternal Demographics**				
Age at delivery (y)	33.1 (29.4, 36.5)	33.1 (29.4, 36.6)	32.9 (30.5, 35.3)	0.92
Hispanic ethnicity	29 (23.0)	22 (21.4)	7 (30.4)	0.41
Non-white race	67 (53.2)	56 (54.4)	11 (47.8)	0.65
Public Insurance	28 (22.2)	22 (21.4)	6 (26.1)	0.59
Pre-pregnancy BMI^c^	24.7 (21.4, 29.1)	24.3 (21.1, 28.6)	26.4 (23.8, 31.0)	0.08
Pre-pregnancy depression or anxiety	26 (20.6)	22 (21.4)	4 (17.4)	0.78
On antidepressants in pregnancy	5 (4.0)	4 (3.9)	1 (4.3)	1.00
Chronic hypertension	16 (12.7)	12 (11.7)	4 (17.4)	0.49
Pre-gestational diabetes	5 (4.0)	4 (3.9)	1 (4.3)	1.00
Nulliparous	78 (61.9)	63 (61.2)	15 (65.2)	0.82
Any first trimester drug/alcohol use	4 (3.2)	3 (2.9)	1 (4.3)	0.57
Timing of EPDS score (weeks postpartum)	6 (6,7)	6 (6,7)	6 (4,6)	0.11
**Baseline Cardiac Factors**				
Acquired cardiac disease	66 (52.4)	56 (54.4)	10 (43.5)	0.65
Congenital cardiac disease	59 (46.8)	47 (45.6)	12 (52.2)	0.37
WHO Classification of CV Risk^d^				
I	45 (35.7)	39 (37.9)	6 (26.1)	0.17
II	46 (36.5)	38 (36.9)	8 (34.8)	
III	20 (15.9)	12 (11.7)	8 (34.8)	
IV	9 (7.1)	8 (7.8)	1 (4.3)	
Anticoagulation with heparin or enoxaparin	40 (31.7)	27 (26.2)	13 (56.5)	**0.007**
**Obstetric Factors**				
Antepartum hospitalization	34 (27.0)	26 (25.4)	8 (34.8)	0.44
Preterm birth <37 weeks	31 (24.6)	23 (22.3)	8 (34.8)	0.28
Preeclampsia	17 (13.5)	12 (11.7)	5 (21.7)	0.20
Gestational diabetes	19 (15.1)	15 (14.6)	4 (17.4)	0.75
General anesthesia	3 (2.4)	2 (1.9)	1 (4.3)	0.46
Blood transfusion	2 (1.6)	0 (0.0)	2 (8.7)	**0.032**
Postpartum readmission	10 (7.9)	8 (7.8)	2 (8.7)	1.00
Breastfeeding	118 (93.7)	98 (95.1)	20 (87.0)	0.16
Any maternal-neonatal separation postpartum^e^	41 (32.5)	29 (28.2)	12 (52.2)	**0.047**
Composite adverse maternal cardiac event during delivery admission^f^	18 (14.3)	14 (13.6)	4 (17.4)	0.74
Intrapartum cardiac/thrombotic event	10 (7.9)	7 (6.8)	3 (13.0)	0.39
Postpartum cardiac/thrombotic event	11 (8.7)	9 (8.7)	2 (8.7)	1.00
Composite adverse neonatal event^g^	27 (21.4)	19 (18.4)	8 (34.8)	0.09
5-minute Apgar <7^h^	4 (3.2)	2 (1.9)	2 (8.7)	0.15
Small for gestational age at birth	14 (11.1)	9 (8.7)	5 (21.7)	0.13
Neonatal death before discharge	2 (1.6)	1 (1.0)	1 (4.3)	0.33

a Data are shown as N (column %) 140 or median (Q1, Q3).

b Wilcoxon rank-sum test was used for continuous variables and Fisher’s exact test used for categorical variables.

c 18 missing values.

d 1 missing value.

e Maternal-neonatal separation during the delivery admission determined based on postpartum location. This includes Maternal telemetry or ICU and NICU admission, Maternal telemetry or ICU and no NICU, or Maternity unit & NICU admission.

f Composite adverse maternal cardiac event includes any intrapartum or postpartum cardiac event or cardiac intervention (cardioversion or other intervention). Any intrapartum cardiac/thrombotic event includes pulmonary edema, chest pain, hypotension, arrhythmia, cardiac failure, thrombotic event, hypoxia with O_2_ saturation persistently below 94%, myocardial infarction, coronary artery dissection, deep vein thrombosis, pulmonary embolism, or cerebrovascular accident. Any postpartum cardiac/thrombotic event includes pulmonary edema, decreased cardiac function, chest pain, arrhythmia, cardiomyopathy or failure, thrombotic event, hypoxia, myocardial infarction, coronary artery dissection, deep vein thrombosis, pulmonary embolism, cerebrovascular accident, organ failure, aortic dissection, or unstable angina.

g Composite neonatal adverse event includes 5-minute APGAR less than 7, small for gestational age at birth, NICU admission, or neonatal death before discharge.

h 3 missing values.

**Table 2:** Longitudinal follow up survey results among English-speaking patients with cardiac disease and a postpartum depression screen result (N=29).

Survey responses	Negative depression screen^a^ N=23	Positive depression screen N=6	P-value^b^
**Survey responder demographics**			
Age at delivery >35	32.2 (29.6, 35.1)	33.5 (32.1, 35.0)	0.62
Gestational age at delivery	37.4 (36.2, 39.3)	35.6 (31.9, 38.1)	0.17
Hispanic ethnicity	4 (17.4)	3 (50.0)	0.13
Non-White race	9 (39.1)	2 (33.3)	1.00
Public Insurance	4 (17.4)	2 (33.3)	0.58
Median family income quartile by zip code^c^			
0–25^th^: $44,124–105,854	5 (23.8)	2 (33.3)	0.55
26–50^th^: $105,855–133,449	5 (23.8)	2 (33.3)	
51–75^th^: $135,604–153,563	6 (28.6)	0 (0.0)	
76–100^th^: $153,564–199,574	5 (23.8)	2 (33.3)	
Congenital cardiac disorder	15 (65.2)	5 (83.3)	0.63
Cardiologist established prior to index pregnancy^c^	20 (87.0)	3 (50.0)	0.08
Nulliparous at time of index pregnancy	18 (78.3)	4 (66.7)	0.61
**Clinical updates since delivery**			
Years since index delivery	2.7 (2.0, 3.6)	1.8 (1.4, 3.1)	0.32
Formally diagnosed with anxiety or depression since index delivery	4 (17.4)	3 (50.0)	0.13
Quality of life score (5=very good, 1=very bad)	5 (4.0,5.0)	4 (3.3, 4.8)	0.16
Activity level^d^			
Sedentary or mild (<=5,000 steps a day or exercise up to once a week)	9 (40.9)	4 (66.7)	0.50
Moderately or very active (at least 5,000 steps a day or exercise > once a week)	13 (59.1)	2 (33.3)	
Cardiac-related hospitalization or complication since delivery	2 (8.7)	2 (33.3)	0.18
Valve replacement or repair	0	1 (16.7)	
Heart failure	1 (4.3)	0	
Pacemaker change & Sinus node ablation	1 (4.3)	0	
Open heart surgery	0	1 (16.7)	
Visit to cardiologist since delivery	17 (73.9)	6 (100)	0.30
Visit to primary care physician since delivery	20 (87.0)	3 (50.0)	0.08
Pregnancy since delivery	5 (21.7)	3 (50.0)	0.31
Planning to get pregnant again			
Yes	5 (21.7)	0 (0.0)	0.68
Uncertain	6 (26.1)	2 (33.3)	
No	12 (52.2)	4 (66.7)	
**Neonatal Outcomes**			
Child started walking	20 (87.0)	5 (83.3)	1.00
If yes, at what age? (months)	13 (11.8, 13.3)	12 (12.0, 15.0)	0.76
Child started talking	21 (91.3)	5 (83.3)	0.52
If yes, at what age? (months)	12 (6.8, 15.3)	12 (9.0, 22.0)	0.61
Child development problems	2 (8.7)	2 (33.3)	0.18
Speech	1	1	
Infantile Spasms	0	1	
Autism, Cerebral Atrophy, Epilepsy	1	0	

a Data are shown as N (column %) or median (Q1, Q3).

b Wilcoxon Rank Sum test was used for continuous variables and Fisher’s exact test used for categorical variables.

c 2 missing values.

d 1 missing value.
